# Plan de cuidados de enfermería para la prevención de úlceras por presión secundarias a la posición prono en pacientes COVID-19

**DOI:** 10.15649/cuidarte.2234

**Published:** 2021-09-13

**Authors:** Lyda Z. Rojas, Liliana Andrea Mora Rico, Jennifer Vanesa Acosta Barón, Luisa Yaneth Cristancho Zambrano, Yurley Dayanna Valencia Barón, Juliana Alexandra Hernández Vargas

**Affiliations:** 1 Fundación Cardiovascular de Colombia, Grupo de Investigación y Desarrollo de Conocimiento en Enfermería GIDCEN, Floridablanca, Colombia. Email: lydarojas@fcv.org Autor de Correspondencia. Fundación Cardiovascular de Colombia Colombia lydarojas@fcv.org; 2 Hospital Internacional de Colombia, Piedecuesta, Colombia. Email: lillianamora@fcv.org Hospital Internacional de Colombia Piedecuesta Colombia lillianamora@fcv.org; 3 Hospital Internacional de Colombia, Piedecuesta, Colombia. Email: jenniferacosta@fcv.org Hospital Internacional de Colombia Colombia jenniferacosta@fcv.org; 4 Hospital Internacional de Colombia, Piedecuesta, Colombia. Email: luisacristancho@fcv.org Hospital Internacional de Colombia Colombia luisacristancho@fcv.org; 5 Fundación Cardiovascular de Colombia, Centro de Investigaciones, Floridablanca, Colombia. Email: yurleyvalencia@fcv.org Fundación Cardiovascular de Colombia Floridablanca Colombia yurleyvalencia@fcv.org; 6 Cuenta de Alto Costo, Fondo Colombiano de Enfermedades de Alto Costo, Bogotá, Colombia. Grupo de Investigación y Desarrollo de Conocimiento en Enfermería GIDCEN. Email: jhernandez@cuentadealtocosto.org Fondo Colombiano de Enfermedades de Alto Costo Bogotá Colombia jhernandez@cuentadealtocosto.org

**Keywords:** Prevención & control, Úlcera por presión, Posición prona, Infecciones por Coronavirus, Pandemias., Prevention & control, Pressure Ulcer, Prone Position, Coronavirus infections, Pan- demics., Prevencão & controle, Lesão por Pressão, Decúbito Ventral, Infecções por coronavirus, Pandemias.

## Abstract

**Introducción:**

La posición prono (PP) es una alternativa terapéutica ampliamente recomendada e implementada en los pacientes con COVID-19. Sin embargo, aunque es un procedimiento no invasivo, es complejo y se asocia con eventos adversos como las úlceras por presión (UPP). Nuestro objetivo es proponer un plan de cuidados de enfermería basado en el lenguaje estandarizado NANDA-I, NIC, NOC para la prevención de las UPP secundarias a la PP en la enfermedad de COVID-19.

**Síntesis del contenido:**

En los pacientes con COVID-19, además de factores de riesgo propios del paciente como la edad avanzada y la presencia de comorbilidades, la PP contribuye a la presencia de los diagnósticos de enfermería de riesgo de úlcera por presión [00249], de deterioro de la integridad cutánea [00047] y tisular [00248]. Por su parte, la intervención de enfermería prevención de úlceras por presión [3540], es clave para minimizar el desarrollo de esta complicación, mejorar la calidad de la atención y el pronóstico en este tipo de pacientes. Finalmente, para determinar la efectividad del cuidado de enfermería se proponen los resultados NOC consecuencias de la inmovilidad: fisiológicas [0204] e integridad tisular: piel y membranas mucosas [1101].

**Conclusión:**

La PP es una terapia coadyuvante recomendada para el manejo de los pacientes con COVID-19 críticamente enfermos, debido a que optimiza la función pulmonar, sin embargo está asociada a eventos adversos como las UPP. Este artículo presenta recomendaciones basadas en una revisión narrativa para facilitar la implementación de cuidados de enfermería preventivos que reduzcan su frecuencia en esta población.

## Abstract (en)

**Introduction:**

Prone position (PP) is a therapeutic alternative widely used and recommended in patients with COVID-19. Although PP is a non-invasive procedure, it is complex and could be associated with complications such as the development of pressure ulcers (PU). We aimed to propose a standardized nursing care plan in terms of NANDA-International, NIC (Nursing Interventions Classification) and NOC (Nursing Outcomes Classification) to prevent PU secondary to the PP in people with COVID-19.

**Content synthesis:**

In patients with COVID-19, in addition to risk factors such as advanced age and the presence of comorbidities, PP contributes to the presence of pressure ulcer risk nursing diagnoses [00249], of deterioration of skin [00047] and tissue [00248] integrity. On the other hand, the nursing intervention for the prevention of pressure ulcers [3540], due to the specificity and scientific basis of its activities, is key to minimize the development of this complication, improve the quality of care and the prognosis in this type of patients. Finally, to evaluate the effectiveness of nursing care, we propose the nursing results (NOC): consequences of immobility: physiological [0204] and tissue integrity: skin and mucous membranes [1101].

**Conclusion:**

PP is a recommended adjunctive therapy for the management of critically ill COVID-19 patients due to its benefits to improve lung function. However, it is associated with adverse effects such as PU. This article presents recommendations based on a narrative review for a better implementation of preventive nursing care that reduces the frequency of PU in this population.

## Resumo

**Introdução:**

: A posição prona (PP) é uma alternativa terapéutica amplamente recomendada e implementada em pacientes com COVID-19. No entanto, embora seja um procedimento não invasivo, é complexo e está associado a eventos adversos, como úlceras por pressão (UP). Nosso objetivo é propor um plano de cuidados de enfermagem baseado na linguagem padronizada NANDA-I, NIC, NOC para a prevenção de UP secundárias à PP na doença COVID-19.

**Síntese de conteúdo:**

Em pacientes com COVID-19, além dos próprios fatores de risco do paciente, como idade avançada e presença de comorbidades, a PP contribui para a presença de diagnósticos de enfermagem de risco de úlcera por pressão [00249], de deterioração da pele [00047] e do tecido Integridade. Por sua vez, a intervenção de enfermagem na prevenção de úlceras por pressão [3540] é fundamental para minimizar o desenvolvimento desta complicação, melhorar a qualidade da assistência e o prognóstico neste tipo de paciente. Por fim, para determinar a eficácia da assistência de enfermagem, são propostos os resultados da NOC, consequências da imobilidade: fisiológicas [0204] e integridade do tecido: pele e mucosas [1101].

**Conclusão:**

PP é uma terapia adjuvante recomendada para o tratamento de pacientes graves com COVID-19, pois otimiza a função pulmonar, porém está associada a eventos adversos, como UP. Este artigo apresenta recomendações baseadas em revisão narrativa para facilitar a implementação de cuidados preventivos de enfermagem que reduzam sua frequência nesta população.

## Introducción

La característica principal de la enfermedad grave por coronavirus 2019 (COVID-19) es la lesión pulmonar aguda manifestada en el síndrome de dificultad respiratoria aguda (SDRA)1 cuya prevalencia oscila entre el 20-41%(2). La posición prono (PP) se indica entre el 16%(3) y el 33%(4) de las personas con SDRA para manejar la lesión pulmonar asociada a la ventilación y promover la oxigenación en los casos graves(1),(5),(6). En la PP el paciente está acostado horizontalmente con la parte frontal del cuerpo hacia abajo y la cabeza ubicada neutralmente; es diferente de la pronación (es decir, la rotación del antebrazo de manera que la palma de la mano quede hacia abajo) y opuesta a la posición supina(7).

La PP está indicada en cirugías de columna, cuello, neurocirugía, colorrectales, vasculares y más recientemente, para el manejo de los pacientes con COVID-19(5). Los principales mecanismos por los que la PP mejora la condición de los pacientes con SDRA son: mejorar la captación en las regiones pulmonares dorsales, aumentar el volumen pulmonar al final de la espiración y la elasticidad de la pared torácica, disminuir el shunt alveolar y optimizar el volumen corriente(8).

Según los resultados de dos metaanálisis, la PP se asoció con una disminución de la mortalidad, aunque no fue estadísticamente significativa(9),(10), sin embargo, en los análisis de subgrupos se evidenció que la PP disminuyó el riesgo de morir en el 42% de los pacientes ventilados con volumen corriente bajo; de éstos, el 40% se encontraba en pronación prolongada, el 51% en instauración de la PP antes de las 48 horas de evolución de la enfermedad y el 49% en hipoxemia(9). Asimismo, redujo la mortalidad en 26% en los pacientes con 12 horas o más de duración de la PP y en aquellos con SDRA moderado a severo y la relación PaO_2_/FiO_2_ al día cuarto fue significativamente mayor en el grupo de PP en comparación con el de posición supina (diferencia de medias: 23,5; IC 95% 12,4 a 34,5)(10).

Debido a estos beneficios y a la buena respuesta a la ventilación en los pacientes críticos en PP, su uso se ha incrementado como alternativa terapéutica en el manejo del COVID-19(11), llevando a que sea recomendada internacionalmente en las guías de práctica clínica de cuidados intensivos(12),(13).

Sin embargo, la PP tiene algunos aspectos a considerar para su implementación. Aunque es un procedimiento no invasivo, es complejo y puede generar importantes eventos adversos que requieren de equipos capacitados para su prevención. Adicionalmente, es una tarea agotadora y demandante para el personal de las unidades de cuidados intensivos sometido a condiciones de estrés6, que sumadas a las particularidades de la pandemia, como la incertidumbre constante sobre los recursos, las capacidades y los riesgos que enfrentan los trabajadores de la salud, así como la exposición al sufrimiento, la muerte y las amenazas a su propia seguridad, incrementan la carga laboral, especialmente del personal de enfermería(14).

Por otra parte, la PP se asocia con complicaciones como el desarrollo de úlceras por presión (UPP), siendo el evento adverso más frecuente (34%), seguido de la neumonía asociada a la ventilación mecánica (21%), la obstrucción del tubo orotraqueal (15%), la extubación accidental (11%), la pérdida del acceso venoso (11%), el neumotórax (6%) y el desplazamiento del tubo orotraqueal (4%)(9). Particularmente, en los pacientes con COVID-19 ventilados en PP, la prevalencia de las UPP oscila entre el 48%(15) y el 61%(16). En términos de su localización, se observó una mayor frecuencia en el mentón, la frente, los pómulos y la nariz(16) y en cuanto a su severidad, las lesiones grado II son las más comunes (~64%)(17),(18).

Se han descrito diversos factores asociados con el desarrollo de UPP secundarias a la PP en comparación con la posición supina, los cuales incrementan hasta 3,3 veces el riesgo de UPP, con mayor afectación del área facial como se expuso previamente(19)-(22). Estos factores son similares a los descritos en otras poblaciones críticamente enfermas que requieren PP dentro del plan de manejo, incluyendo: a) la inmovilidad, la mala percepción y respuesta sensorial y, b) la susceptibilidad y tolerancia individual donde las características de la piel, la pobre perfusión y nutrición, la diabetes, el exceso de humedad y la albúmina disminuida son factores contribuyentes(23). Además de lo anterior, un claro factor de riesgo es el promedio de duración de la PP, que se relaciona directamente con la aparición y la severidad de las UPP en las personas con COVID-19(15)-(17), incrementando sustancialmente el riesgo, incluso desde el tercer día(15).

En relación con el tratamiento de las UPP, este es largo y genera una carga económica importante para el sistema de salud. En los Estados Unidos, se gastan aproximadamente $11 mil millones de dólares anuales derivados de su cuidado y, entre $500 y $70.000 en una sola herida(24). Aunque no se ha realizado una estimación del incremento en los costos asociados al cuidado de las UPP en los pacientes con COVID-19 se hipotetiza un impacto importante, a tal punto que el cuidado de las heridas ha cobrado mayor relevancia, pasando de ser un área subestimada a un eje clave dentro de las guías y protocolos de atención, incluyendo las relacionadas con los cuidados de enfermería.

En este sentido, además del control de los síntomas y el tratamiento de las complicaciones secundarias a la COVID-19, se deben implementar medidas preventivas frente a las UPP derivadas de la PP(21), con el fin de disminuir la alta tasa de incidencia de este evento adverso producto de la atención en salud de los pacientes con SDRA.

En consideración con lo expuesto, el objetivo de este artículo es proponer un plan de cuidados de enfermería basado en los lineamientos establecidos en NANDA Internacional *(NANDA-I)*, NIC (*Nursing Interventions Classification*) y NOC (*Nursing Outcomes Classification*) para la prevención de las UPP secundarias a la PP en la enfermedad de COVID-19, con el fin de orientar la implementación y evaluación de intervenciones basadas en una revisión narrativa que contribuyan a una mayor seguridad y calidad de la atención, teniendo en cuenta que las UPP se consideran un evento adverso producto de fallas en la atención de enfermería.

### Factores de riesgo y diagnósticos de enfermería en las UPP

Los pacientes con COVID-19 hospitalizados en unidades de cuidado crítico presentan factores de riesgo que favorecen el desarrollo de las UPP como los extremos de la vida (edad avanzada 60,5 ± 14,5 años), el estado nutricional desequilibrado (sobrepeso/obesidad, con una mediana del índice de masa corporal de 30,5, Q1=26,6; Q3=36,2), los agentes químicos/secreciones (náuseas o vomito 17,7%, diarrea 20,8%), las comorbilidades (hipertensión 59,7%, diabetes 38,9%, enfermedades pulmonares crónicas 24,0%) y la hipoperfusión (mediana PaO_2_/FiO_2_ 124, Q1=86; Q3=188) generada por la disnea (74,9%), la fatiga (32,5%) o el tratamiento con vasopresores (48,3%)(25).

De igual forma, los casos graves tienen menores niveles de hemoglobina comparados con los moderados (diferencia de medias ponderada de hemoglobina -4,08 g/L; IC del 95% -5,12; -3,05)(26) y fricción con la superficie/disminución de la movilidad (posición prono 27,0% y 38,5%)(25), (27) así como hipertermia (fiebre mediana 38,1°C, Q1=37,3; Q3=38,9)(25).

Teniendo en cuenta estas características y la coincidencia de estos factores de riesgo con los enunciados en NANDA-I, se proponen tres diagnósticos de enfermería para prevenir las UPP secundarias a la PP en la enfermedad de COVID-19: “riesgo deterioro de la integridad cutánea [00047]” definido como la susceptibilidad a una alteración de la epidermis y/o de la dermis que puede comprometer la salud, “riesgo de deterioro de la integridad tisular [00248]” definido como susceptible a una lesión de la membrana mucosa, córnea, sistema intertegumentario, fascia muscular, músculo, tendón, hueso, cartílago, cápsula de la articulación y/o ligamento que puede comprometer la salud y, el “riesgo de úlcera por presión [00249]” definido como la susceptibilidad a lesiones localizadas de la piel y/o tejido subyacente por lo general en un relieve óseo como resultado de la presión, o la presión en combinación con el cizallamiento(28).

### Cuidados de enfermería para la prevención de las UPP

Para la selección de las intervenciones y los resultados de enfermería se tuvo en cuenta los vínculos entre las clasificaciones NANDA-I (2018-2020), NOC (6ª edición) y NIC (7ª edición), identificados con la herramienta NNN- Consult(28). Se priorizó la intervención “Prevención de úl- ceras por presión [3540]” definida como la prevención de la formación de UPP en un individuo con alto riesgo de desarrollarlas, dada su robustez para atender los factores de riesgo identificados en los tres diagnósticos de enfer- mería previamente propuestos y el alcance general de las actividades que sugiere. Para fundamentar las actividades descritas en dicha intervención se realizó una revisión narrativa, para lo cual se hicieron búsquedas en las bases de datos PUBMED, EMBASE y CINALH, combinando los siguientes términos libres: “Pressure Ulcers ” OR “ Pressure Sore ” OR “ Pressure Injuries ” AND “ Prone Position ” AND “ COVID-19”. Se seleccionaron los artículos que permitieron justificar las actividades seleccionadas (Tabla 1).

**Tabla 1:** Intervención de enfermería: Prevención de úlceras por presión (3540)

Actividades (NIC) (28)	Referencia
Utilizar una herramienta de valoración de riesgo establecida para valorar los factores de riesgo del individuo (escala de Braden).	La evaluación del riesgo de UPP debe efectuarse antes que el paciente sea candidato para la PP y cuando retorne a la posición de supino según el plan de cuidados(29).
	La evaluación del riesgo de UPP se considera una buena práctica de cuidado. No obstante, la frecuencia con la que debe realizarse no está bien definida; depende del juicio clínico y el riesgo inherente al paciente, dado por el pronóstico clínico y el número de dispositivos de cuidado instalados(30). No se ha descrito una escala específica para evaluar el riesgo de UPP en esta población.
	Sin embar- go, los resultados de un meta-análisis sugieren que las escalas de Braden, Norton y Waterlow tienen una sensibilidad, especificidad y valores predictivos similares y pueden aplicarse en contextos de cuidado crítico(31),(32). En los casos en los que se observe la úlcera por presión, la valoración del riesgo debe continuarse con el Sistema Internacional de Clasificación de las Úlceras por Presión del Panel Asesor Nacional de UPP (NPIAP, por su sigla en inglés) y del Panel Asesor Europeo de UPP (EPUAP, por su sigla en inglés)(33).
	Se recomienda realizar más de dos evaluaciones del riesgo de UPP al día en aquellos pacientes con múltiples dispositivos médicos, cambios de líquidos frecuentes y con signos de edema localizado o generalizado(33).
Registrar el estado de la piel durante el ingreso y luego a diario.	La vigilancia de las características de la piel, debe incluir la valoración de las mucosas, puesto que el eritema no blanqueable es poco visible. Es importante realizar una inspección detallada de la mucosa oral debido a que lesiones aparentemente superficiales pueden ser más profundas y estar asociadas a una mayor extensión (33)
Eliminar la humedad excesiva en la piel causada por la transpiración, el drenaje de heridas y la incontinencia fecal o urinaria.	Es necesario mantener la piel limpia e hidratada en un nivel óptimo, evitando el exceso de hume- dad o resequedad que pueden generar maceración(30).
	La vigilancia del nivel de hidratación de la piel es fundamental debido a que la mayoría de pacientes con SDRA son llevados a un balance hídrico negativo(33).
	La frecuencia de limpieza de la piel debe ser determinada de manera individual para no afectar la hidratación de la piel que tiene una función de barrera natural contra las consecuencias de la fricción(33).
Vigilar las fuentes de presión y de fricción.	La cara y aquellas áreas corporales que soportan mayor peso como la región mamaria, el tórax, las clavículas, la cresta ilíaca y las rodillas han sido descritas en la literatura como las de mayor riesgo de UPP en este tipo de pacientes(4),(20),(33),(35) ver Figura 1.
	La frente, los pómulos y la barbilla son las zonas más susceptibles al desarrollo de UPP en los pacientes sometidos a la PP. Por lo anterior, se debe extremar su valoración periódica y la liberación de las fuentes de presión o fricción(36).
	En los hombres, es particularmente importante vigilar que no existan zonas de presión en el área genital(35).
Inspeccionar la piel de las prominencias óseas y demás puntos de presión al cambiar de posición al menos una vez al día.	La presencia de dispositivos médicos (circuitos de infusión, monitorización de signos vitales o sondajes) es un factor de riesgo para los pacientes en la PP debido a la presión sostenida que ejercen sobre ciertas zonas del cuerpo, por lo que se debe evitar el contacto estrecho con los mismos(33),(37).
	En las personas con tubos endotraqueales, los sitios anatómicos que se han asociado con una mayor frecuencia de UPP son los labios y la boca(35),(38).
	En las personas con sondas nasogástricas, la valoración de la nariz, las narinas y el puente nasal es fundamental debido a que estas lesiones son más frecuentes en dichos sitios anatómicos(35),(38).
Evitar el agua caliente y utilizar un jabón suave para el baño.	Mantener la piel limpia e hidratada utilizando productos con pH balanceado es prioritario. Sin embargo, la evidencia sobre el tipo de hidratantes que se deben emplear es inconsistente(29).
	Tradicionalmente, la limpieza de la piel se ha realizado con agua y jabón. No obstante, existe evidencia de su impacto desfavorable sobre el balance del pH. Al respecto, otros productos que contienen surfactantes pueden proveer una mayor protección a la piel(33),(39),(40).
Aplicar barreras de protección, como cremas o compresas absorbentes, para eliminar el exceso de humedad, según corresponda.	El uso de limpiadores con pH entre 4,0 y 7,0 (ligeramente ácido a neutro) ha sido costoefectivo para reducir la resequedad, el eritema, la irritación de la piel y, en general la incidencia de las UPP(33),(39),(40).
	La aplicación de ácidos grasos hiperoxigenados es efectiva en la prevención de las UPP relacionadas con la ventilación mecánica en este grupo de pacientes(41).
	El uso profiláctico de apósitos como hidrocoloides, películas transparentes y de silicona han sido eficaces para disminuir la presión y la degradación de la piel de la cara, principalmente(19),(29) ver Figura 2.
	El uso de hidrocoloides y películas transparentes reduce hasta en un 50% la incidencia de las UPP en las áreas faciales(42).
Colocar al paciente en posición ayudándose con almohadas para elevar los puntos de presión encima del colchón.	El uso profiláctico de apósitos en las zonas circundantes a la instalación de dispositivos médicos como el circuito de ventilación mecánica, sondas nasogástricas o sistemas de infusión de medicamentos es recomendable para disminuir la presión y la fricción(5),(30),(33),(38).
	Teniendo en cuenta que la presencia de tubos endotraqueales y sondas nasogástricas incrementa el riesgo de UPP, su reposicionamiento lateral ha demostrado ser beneficioso para la prevención de lesiones de tejidos blandos(33).
Utilizar mecanismos en la cama (badana) para proteger al paciente.	El uso de superficies de apoyo adecuadas y almohadas es crucial para prevenir la deformación del tejido y mejorar la perfusión tisular(43).
	El uso de sistemas de soporte para la cabeza en la PP reduce significativamente la incidencia de las UPP(43).
	Con respecto a los dispositivos para dar soporte a la cabeza, aquellos en forma de anillo o dona deben evitarse debido a que causan un mayor cizallamiento y presión en la superficie y capas internas de la piel, incrementando el riesgo de UPP(44),(45).
	Con respecto a los dispositivos para dar soporte a la cabeza, aquellos en forma de anillo o dona deben evitarse debido a que causan un mayor cizallamiento y presión en la superficie y capas internas de la piel, incrementando el riesgo de UPP(44),(45).
Evitar mecanismos de tipo flotador para la zona sacra.	El uso de almohadas para el acojinamiento de las estructuras faciales ha sido eficiente para disminuir la incidencia de las UPP(46). El desplazamiento y la deformidad de la piel y los tejidos blandos no dependen de la dureza de los colchones sino de su forma. Una mayor inclinación del colchón incrementa el desplazamiento de la superficie de la piel y el riesgo de UPP(47).
	Como parte del cuidado de enfermería, es prioritario identificar las actividades específicas para ciertas áreas corporales expuestas a un alto riesgo(5):
	**Ocular:** además de la lesión corneal, la PP puede ocasionar pérdida de la visión debida principalmente a isquemia del nervio óptico o síndrome compartimental. Para su prevención se recomienda el uso de reposacabezas para liberar la presión directa sobre las estructuras oculares(18),(46),(48).
	La aplicación de lubricante y cerrar los párpados con cinta adhesiva también es recomendable(18).
	Adicionalmente, la posición de la cama en Trendelenburg es útil para reducir la presión ocular y el edema(48).
	**Tórax:** los cables de los dispositivos de monitoreo hemodinámico o de los sistemas de infusión deben reposicionarse en la zona de la espalda o lateralmente en las extremidades con el fin de evitar su presión excesiva.
	Se debe garantizar que todas las líneas centrales, arteriales o cánulas estén perfectamente aseguradas y en lo posible, fijadas lejos del área del tórax(18).
	Genitales y extremidades inferiores: Las sondas vesicales o dispositivos para la eliminación fecal deben posicionarse fuera de la zona que está en contacto estrecho con la cama(18). Se recomienda el uso de apósitos profilácticos o férulas en la patela o el área pretibial(18). El uso de dispositivos para mejorar la posición de las zonas de alto riesgo, sumado a los cambios de posición son altamente
	recomendables para disminuir los puntos de presión en la cara y otras áreas corporales(29).
	Las superficies de soporte funcionan para redistribuir la presión y el cizallamiento en
	la piel, promoviendo una mejor perfusión tisular y aliviando las deformaciones del tejido(49),(50).
Darse la vuelta continuamente cada 1-2 horas, según corresponda.	Los cambios de posición habituales con una frecuencia determinada según la evaluación del riesgo, así como el reposicionamiento de los dispositivos médicos son eficientes para disminuir las zonas de presión y evitar la ruptura de la piel y los tejidos blandos(33).
Darse la vuelta con cuidado (p. ej., evitar el cizallamiento) para evitar lesiones en una piel frágil.	El reposicionamiento corporal debe realizarse como mínimo cada dos horas en los pacientes que han sido sometidos a ciclos de 12 horas o más en esta posición(5),(35).
Poner el programa de cambios posturales al lado de la cama, según corresponda.	Al realizar los cambios de posición es fundamental conservar una buena alineación esquelética con el fin de reducir la tensión muscular a un nivel mínimo. El NPIAP recomienda la posición de natación utilizando almohadas para dar soporte a las zonas corporales, alternando la posición de los brazos y la cabeza cada 2 horas para disminuir el riesgo de UPP en los codos y la cara (Figura 3). Se debe garantizar que el tubo endotraqueal no se desplace u obstruya durante el reposicionamiento(5),(18),(51).

NIC: Nursing Intervention Classification.

### Evolución y eficiencia de los cuidados preventivos de las UPP

Para la selección de los resultados se tuvo en cuenta la población del contexto del artículo (pacientes críticos con SDRA secundaria a COVID-19 en posición prona, en unidades de

cuidados intensivos y sedados)(52) y los vínculos NANDA-I, NOC y NIC. Por tanto, sugerimos dos resultados para monitorizar la efectividad de las intervenciones y evitar que se avance a diagnósticos reales de enfermería. El primero, es la “Integridad tisular: piel y membranas mucosas [1101]” definido como la indemnidad estructural y función fisiológica normal de la piel y las membranas mucosas, con los indicadores de: hidratación [110104], perfusión tisular [110111], integridad de la piel [110113] medidos con la escala 01 (Grado de deterioro de la salud o el bienestar) y los indicadores lesiones de la mucosa [110116], eritema [1101121], palidez [110122] evaluados con la escala 14 (Grado de un estado o respuesta negativo a adverso) con opciones de respuesta de grave (1), sustancial (2), moderado (3), leve (4) y ninguno (5)(28). El segundo se denomina “Consecuencias de la inmovilidad: fisiológicas [0204]”**,** definido como la gravedad del compromiso en el funcionamiento fisiológico debido a la alteración de la movilidad física y su indicador más pertinente es el de úlceras por presión [20401](28).

En la Tabla 2 se observa un ejemplo de la operacionalización del indicador úlceras por presión [20401](28) utilizando las escalas de evaluación del riesgo de UPP. Cada parámetro evaluado califica del 1 al 4 (siendo 1 el peor estado 1 y 4 el mejor) y en términos de los resultados de enfermería (Grave:1, Moderado:2, Leve:3 y Ninguno:4), arbitrariamente omitimos la categoría “sustancial” para que coincida con las escalas de medición de los instrumentos para evaluar el riesgo de UPP y así no alterar sus propiedades psicométricas.

**Tabla 2:** Operacionalización del indicador úlceras por presión [20401] utilizando las escalas de evaluación del riesgo

Consecuencias de la inmovilidad: fisiológicas [0204](28)			
Indicador NOC	Parámetro a evaluar	Grave (1)	Moderado (2)	Leve (3)	Ninguno (4)
Úlceras por presión (Escala de Braden)^53^	Percepción sensorial	Complemente limitada	Muy limitada	Ligeramente limitada	Sin limitación
	Exposición a la humedad	Siempre	A menudo	Ocasionalmente	Raramente
	Actividad física	Encamado	En silla	Deambula ocasionalmente	Deambula frecuentemente
	Movilidad cambios posturales	Inmóvil	Muy limitada	Levemente	Sin limitación
	Nutrición	Muy pobre	Probablemente inadecuado	Adecuado	Excelente
	Cizallamiento y roce	Presente	Potencialmente presente	No existe problema aparente	
Indicador NOC	Parámetro a evaluar	Grave (1)	Moderado (2)	Leve (3)	Ninguno (4)
Úlceras por presión (Escala de Norton)^54^	Estado físico general	Muy malo	Malo	Regular	Bueno
	Estado mental	Estuporoso o comatoso	Confuso	Apático	Alerta
	Movilidad	Inmóvil	Muy limitada	Disminuida	Total
	Actividad	Encamado	Sentado	Camina con ayuda	Ambulante
	Incontinencia	Urinaria y fecal	Urinaria o fecal	Ocasional	Ninguno

NOC: Nursing Outcomes Classification.

**Figura 1:**
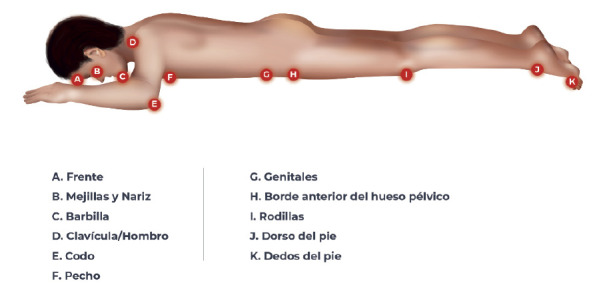
Áreas potenciales de presión en la posición prono

Fuente: propia.

**Figura 2:**
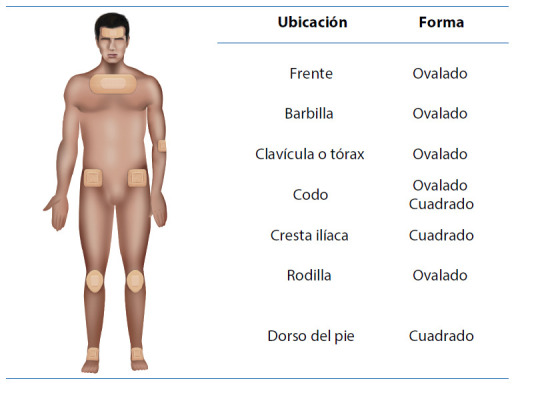
Guía del uso profiláctico de apósitos para disminuir la presión y prevenir las úlceras por presión

Fuente: propia.

**Figura 3:**
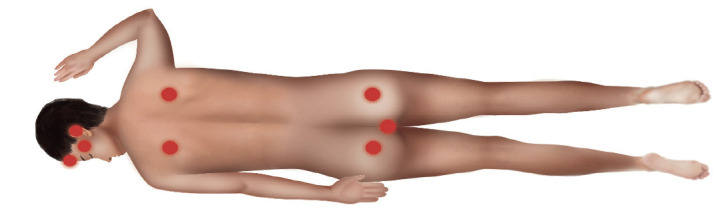
Posición de natación, estilo libre

Fuente: propia. Los puntos de presión de la figura corresponden a la superficie del cuerpo que mira hacia el colchón, previamente descritos en la Figura 1.

### Consideraciones Finales

Se requieren equipos de trabajo capacitados y entrenados tanto para brindar los cuidados relacionados con la PP como para prevenir las UPP, debido a que lo anterior demanda un trabajo más intenso y es todo un desafío para el personal de enfermería. Existe una herramienta para demostrar la competencia de enfermería en la PP de los pacientes y evalúa cinco aspectos: 1) indica las condiciones para colocar al paciente en la PP; 2) declara las contraindicaciones para la PP; 3) prepara al paciente para la PP; 4) ayuda al paciente a colocarse en la PP y garantiza una posición anatómica correcta, y 5) evalúa la respuesta del paciente a la PP(55), la cual podría ser útil a nivel clínico.

En resumen, la PP es una terapia coadyuvante recomendada para el manejo de pacientes con COVID-19 críticamente enfermos con manifestaciones de SDRA grave. Su uso se ha incrementado debido a los beneficios para mejorar la función pulmonar, sin embargo, también se ha asociado a eventos adversos como las UPP y otras complicaciones. Este artículo presenta recomendaciones basadas en la literatura científica para sustentar un plan de cuidados de enfermería dirigido a la implementación de actividades preventivas que reduzcan la frecuencia de las UPP secundarias a la PP en la enfermedad de COVID-19, promoviendo el cuidado integral de calidad. Sin embargo, se debe tener precaución en la interpretación de estas recomendaciones dado que se realizó una revisión narrativa para sustentar el plan de cuidados y, debido al alcance de la misma no se evaluó la calidad metodológica de los artículos, por lo que se requiere de una búsqueda sistemática para definir su nivel de evidencia y grado de recomendación en la práctica clínica de enfermería.
